# Phosphodiesterase inhibitor ameliorates senescent changes of renal interstitial pericytes in aging kidney

**DOI:** 10.1111/acel.14075

**Published:** 2023-12-28

**Authors:** Hyung Duk Kim, Eun Nim Kim, Ji Hee Lim, Yaeni Kim, Tae Hyun Ban, Hajeong Lee, Yon Su Kim, Cheol Whee Park, Bum Soon Choi

**Affiliations:** ^1^ Department of Internal Medicine, Eunpyeong St. Mary's Hospital, College of Medicine The Catholic University of Korea Seoul Republic of Korea; ^2^ Department of Internal Medicine, College of Medicine The Catholic University of Korea Seoul Republic of Korea; ^3^ Department of Internal Medicine, Seoul St. Mary's Hospital, College of Medicine The Catholic University of Korea Seoul Republic of Korea; ^4^ Department of Internal Medicine Seoul National University Hospital Seoul Republic of Korea

**Keywords:** aging, microvascular rarefaction, pentoxifylline, pericytes, phosphodiesterase inhibitors

## Abstract

Pericytes are mesenchymal cells that surround endothelial cells, playing a crucial role in angiogenesis and vessel maturation. Additionally, they are associated with interstitial fibrosis as a major contributor to renal myofibroblasts. In this study, we aim to investigate whether the phosphodiesterase inhibitor, pentoxifylline (PTX), can ameliorate aging‐related functional and histological deterioration in the kidney. We subjected aging C57BL/6 mice, dividing into young, aging, and PTX‐treated aging groups. Renal function, albuminuria, and histological changes were assessed. Interstitial pericytes were assessed by immunohistochemistry analysis. We examined changes in pericytes in elderly patients using human kidney tissue obtained from healthy kidney donors for kidney transplantation. In vitro experiments with human pericytes and endothelial cells were performed. Aging mice exhibited declined renal function, increased albuminuria, and aging‐related histological changes including mesangial expansion and tubulointerstitial fibrosis. Notably, number of pericytes declined in aging kidneys, and myofibroblasts increased. PTX treatment ameliorated albuminuria, histological alterations, and microvascular rarefaction, as well as modulated angiopoietin expression. In vitro experiments showed PTX reduced cellular senescence and inflammation. Human kidney analysis confirmed similar pericyte changes in aging kidneys. The phosphodiesterase inhibitor, PTX preserved microvascular density and improved renal interstitial fibrosis and inflammation in aging mice kidneys. These protective effects were suggested to be associated with the amelioration of pericytes reduction and the transition to myofibroblasts. Additionally, the upregulation of angiopoietin‐1 expression may exert potential impacts. To the best of our knowledge, this is the first report on the changes in renal interstitial pericytes in aging human kidneys.

## Graphical abstract

In this study, pentoxifylline (PTX) treatment demonstrated protective effects in aging mice kidneys, preserving microvascular density and ameliorating interstitial fibrosis and inflammation. These effects were associated with mitigating pericyte reduction, inhibiting myofibroblast transition. And upregulation of angiopoietin‐1 expression may exert potential impacts. This study suggests novel insights into PTX's propective role in renal aging.

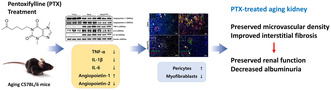

**Abbreviations**
ACR: Albumin‐to‐creatinine ratio
BUN: Blood urea nitrogen
CD31: Cluster of differentiation 31
DKD: Diabetic kidney disease
DM: Diabetes mellitus
DOXO: Doxorubicin
eGFR: Estimated glomerular filtration rate
HTN: Hypertension
IF: Immunofluorescence
IFN‐γ: Interferon‐γ
IL‐1β: Interleukin‐1β
IL‐6: Interleukin‐6
MCP‐1: Monocyte chemoattractant‐1
NG2: Neural‐glial antigen 2
PAS: Periodic acid–Schiff
PBS: Phosphate‐buffered saline
PDE: Phosphodiesterase
PDGFR‐β: Platelet‐derived growth factor receptor‐β
PTX: Pentoxifylline
SA‐β‐gal: Senescence‐associated β‐galactosidase
TNF‐α: Tumor necrosis factor‐α
α‐SMA: α‐smooth muscle actin

## 1 INTRODUCTION

Aging is a natural, continuous, and inevitable biological process of gradual decline of the function of many organ systems (Denic et al., 2016; O'Sullivan et al., 2017). In the kidney, the aging process manifests with decrease in nephron size and number, tubulointerstitial changes, glomerular basement membrane thickening, and glomerulosclerosis (O'Sullivan et al., 2017). The major compartments of the kidney are the glomerulus, tubulointerstitium, and the capillary network. These renal compartments undergo glomerulosclerosis, interstitial fibrosis, tubular atrophy, and microvascular rarefaction during aging and are closely related to each other (Schmitt & Melk, 2017). Recent research has focused mainly on the mechanism involved in each of the aging‐related changes.

Pericytes are extensively branched mesenchymal cells that surround endothelial cells in the capillary bed (Díaz‐Flores et al., 2009). They form an extensive network around the microvasculature throughout the body. They play important role in angiogenesis, vessel maturation, and response to injury (Kramann & Humphreys, 2014). Kidney pericytes maintain microvascular integrity (O'Sullivan et al., 2017) and regulate renal blood flow (Kennedy‐Lydon et al., 2013). Recently, pericytes have emerged as the major contributors to renal myofibroblasts via pericyte‐myofibroblast trans‐differentiation, (Humphreys et al., 2010) and their detachment from capillaries could possibly trigger capillary rarefaction (Kramann et al., 2014). Stefanska et al. (2015) had previously reported the decline in kidney interstitial pericytes as a critical step in the development of peritubular vasculature changes during aging, and the associated fibrosis.

The phosphodiesterase inhibitor pentoxifylline (PTX) has emerged as a renoprotective agent that induces hemodynamic changes in drug‐associated nephropathies and improves renal function (Vadiei et al., 1996). Among all the phosphodiesterase (PDE) isozymes, PTX inhibits PDE 1–5. In accordance with the effect of a selective PDE 3 inhibitor, PTX has been reported to inhibit mesangial cell proliferation both in vitro and in vivo (Lin et al., 2004). PTX was shown to inhibit the secretion of proinflammatory cytokines, such as tumor necrosis factor‐α (TNF‐α), monocyte chemoattractant‐1 (MCP‐1), interleukin‐1β (IL‐1β), interleukin‐6 (IL‐6), and interferon‐γ (IFN‐γ), in the kidneys of experimental animal models (Lin et al., 2004). In experimental models of mesangial proliferative glomerulonephritis and crescentic glomerulonephritis, PTX was confirmed to alleviate interstitial fibrosis (Chen et al., 1999, 2004). In addition, PTX has been reported to exhibit antifibrotic effects by inhibiting extracellular matrix synthesis and myofibroblast differentiation to renal fibroblasts (Strutz et al., 2000).

In this study, we hypothesized that PTX ameliorates functional and/or histological deterioration of aging kidneys by attenuating the decline in the number and myofibroblast transition of pericytes.

## 2 METHODS

### 2.1 Animal model

**FIGURE 1:**
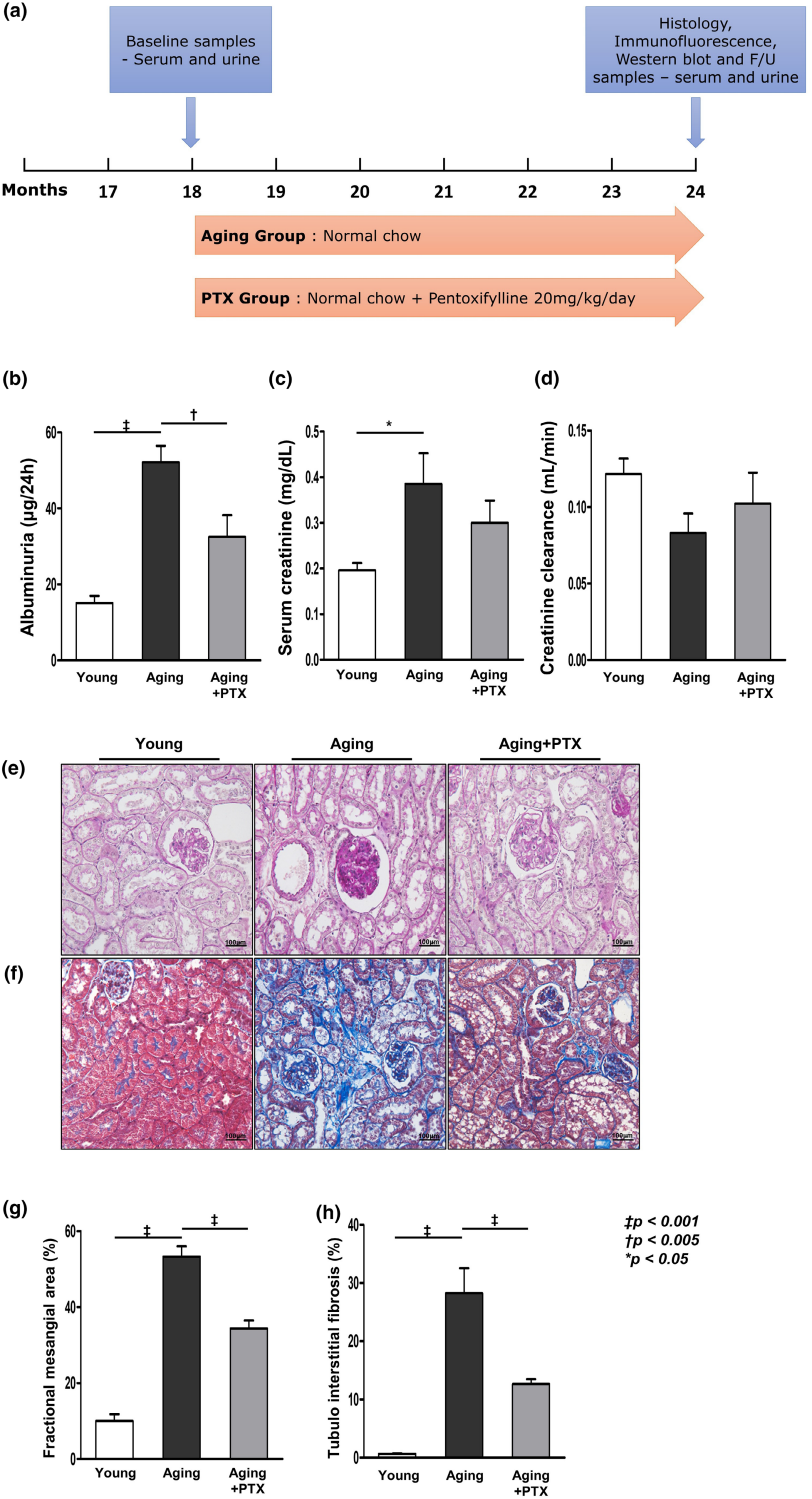
Study protocol and effects of pentoxifylline (PTX) on renal function and aging‐related histological renal injuries. (a) Schematic representation of the study protocol. Baseline samples including serum and urine were obtained at the 18th month. PTX was administered for 6 months at a dosage of 20 mg/kg/day in PTX group. The experimental animals were sacrificed at the 24th month, and serum, urine, and kidney tissue samples were obtained for analysis. Compared to the aging group, PTX group showed (b) lower albuminuria. PTX group showed (c) lower serum creatinine and (d) better creatinine clearance, though not statistically significant. (e) Representative photomicrographs of the periodic acid‐Schiff‐(PAS)‐stained kidney showed less expansion of the mesangial area in PTX group (original magnification, 400×). (f) Representative sections of the Masson's trichrome‐stained kidney showed significantly less tubulointerstitial fibrosis in PTX group (original magnification, 200×). Quantitative assessments of (g) the areas of extracellular matrix in the glomerulus and (h) the areas of tubulointerstitial fibrosis. (*N* ≥ 7 for all experiments. ^‡^ *p* < 0.001, ^†^ *p* < 0.005, *p < 0.05).

The Animal Care Committee of The Catholic University approved the experimental protocol. Aging C57BL/6 mice were purchased from the Korea Research Institute of Bioscience and Biotechnology (Chungcheongbuk‐do, Republic of Korea). The mice were housed in a controlled‐temperature and controlled‐light environment. They were divided into three groups, namely 2‐month‐old group (young group, *n* = 7), 24‐month‐old group (aging group, *n* = 7), and 24‐month‐old PTX‐treated group (PTX group, *n* = 7). PTX group mice were administered water containing PTX (US Pharmacopeial Convention, NJ, USA) at 20 mg/kg/day for 6 months (Figure 1A).

### 2.2 Renal function and albuminuria

The mice were placed in individual metabolic cages (Tecniplast, Gazzada, Italy), with 24‐h access to water and food, to collect urine for subsequent measurements of albumin and creatinine concentrations. Albuminuria (Albuwell M; Exocell, Philadelphia, PA, USA) and urine creatinine (Creatinine Companion; Exocell) levels were measured using ELISA kits. Serum creatinine levels were assessed using i‐STAT system cartridges (CHEM8+; Abbott Point of Care Inc., Abbott Park, IL, USA). Creatinine clearance was calculated using the standard formula (urine creatinine [mg/dL] × urine volume [mL/24 h]/serum creatinine [mg/dL] × 1440 [min/24 h]).

### 2.3 Histological change assessment

Kidney samples were fixed in 4% paraformaldehyde. The tissues were embedded in low‐temperature‐melting paraffin, and 4‐μm‐thick sections were processed and stained with periodic acid–Schiff (PAS) and Masson's trichrome stain. The glomerular volume and mesangial area were determined by examining the PAS‐stained sections, and the relative mesangial area was expressed as the ratio of mesangial/glomerular surface area. Tubulointerstitial fibrosis is defined as a matrix‐rich expansion of the interstitium with tubular dilatation, tubular atrophy, tubular cast formation, and thickening of the tubular basement membrane. Stained tissue samples were assessed, 10 fields per section, and observed under a Leica light microscope DMI 5000B (Leica, Wetzlar, Germany). After analyzing all the sections, the data were assessed using ImageJ software (Wayne Rasband, National Institutes of Health, USA).

### 2.4 Immunofluorescence analysis

The paraffin sections were deparaffinized in xylene and hydrated in ethanol before immunofluorescence (IF). Sections were treated with an antigen‐unmasking solution consisting of 10 mM sodium citrate (pH 6.0) and then washed with phosphate‐buffered saline (PBS). The sections were then incubated with 3% H_2_O_2_ in methanol to block all endogenous peroxidase activity. Nonspecific binding was blocked using 10% normal horse serum. After incubation with the primary antibody in a humidified chamber at 4°C overnight, fluorescent‐labeled secondary antibodies were added, according to the manufacturer's protocol. DAPI (Invitrogen, Carlsbad, CA, USA) was used for nuclear counterstaining. All sections were observed using a laser scanning confocal microscope (Carl Zeiss LSM 700, Oberkochen, Germany) and then assessed using ImageJ software (Wayne Rasband, National Institutes of Health, USA).

### 2.5 Renal fibrosis by IF

To confirm fibrosis of the renal tissues, Collagen I (Abcam, Cambridge, UK; 1:200) and α‐smooth muscle actin (α‐SMA) (Abcam, Cambridge, UK; 1:100) antibodies were used. Sections were stained with Alexa 594‐ and Alexa 488‐conjugated anti‐rabbit secondary antibodies (Invitrogen, Carlsbad, CA, USA), respectively.

### 2.6 Pericyte assessment by IF

Formalin‐fixed kidney sections underwent deparaffinization, heat‐mediated antigen retrieval in citrate buffer, and nonspecific background blocking. Pericytes were identified by the co‐expression of platelet‐derived growth factor receptor‐β (PDGFR‐β) (Abcam, Cambridge, UK; 1:100) and neural‐glial antigen 2 (NG2) (Abcam, Cambridge, UK; 1:200) antigens, followed by incubation with Alexa Fluor 488‐conjugated anti‐rabbit IgG (Thermo Fisher Scientific, MA, USA) and Alexa Fluor 405‐conjugated anti‐mouse IgG (Thermo Fisher Scientific, MA, USA). Perivascular pericytes were identified and evaluated by co‐staining for vascular endothelial cell markers. The vascular endothelial cell marker cluster of differentiation 31 (CD31) (Dianova, Hamburg, Germany; 1:50) antibody was used in combination with Alexa Fluor 594‐conjugated anti‐rat IgG (Thermo Fisher Scientific, MA, USA). In addition, pericyte‐derived myofibroblasts were identified through co‐staining with α‐SMA (Abcam, Cambridge, UK; 1:100). After incubation with α‐SMA antibody, labeled with Alexa Fluor 594‐conjugated anti‐goat IgG (Thermo Fisher Scientific, MA, USA).

### 2.7 Pericytes in human kidney

Human kidney tissue was obtained from healthy kidney donors for kidney transplantation by performing a zero‐time biopsy at Seoul National University Hospital. A zero‐time biopsy was performed immediately before allograft implantation during kidney transplant surgery. All donors were confirmed to have normal renal function as per pretransplant evaluation. Twelve donors in their 20s were assigned to the young group, and 12 donors in their 60s were assigned to the aging group. Similar to that in the animal tissue, IF staining was performed to confirm the changes in the pericytes in human tissue. The perivascular pericytes were identified through triple co‐staining of PDGFR‐β, NG2, and CD31, and their transformation into pericyte‐derived myofibroblasts was confirmed through triple co‐staining of PDGF‐β, NG2, and α‐SMA compared between the two groups. The study was approved by the Institutional Review Board of Seoul National University Hospital (C‐1810‐095‐980) and was conducted in accordance with the principles of the Helsinki Declaration II. Antibodies and methods were the same as those used in animal staining.

### 2.8 Western blot analysis

Total protein was extracted from renal cortical tissues using the PRO‐PREP Protein Extraction Solution (iNtRON Biotechnology, Gyeonggi‐Do, Republic of Korea), according to the manufacturer's instructions. Western blot analysis was performed using the following antibodies: angiopoietin‐1 (Proteintech, Rosemont, IL, USA; 1:2000), angiopoietin‐2 (Proteintech, Rosemont, IL, USA; 1:2000), TNF‐α (Abcam, Cambridge, UK; 1:500), IL‐6 (Huabio, MA, USA; 1:2000), IL‐1β (MyBioSource, CA, USA; 1:3000), β‐actin (Sigma, St Louis, MO, USA; 1:10000), anti‐rabbit IgG HRP‐linked secondary antibody (Cell Signaling Technology, MA, USA; 1:2000), and anti‐mouse IgG HRP‐linked secondary antibody (Cell Signaling Technology, MA, USA; 1:2000).

### 2.9 Cell culture and in‐vitro experiments

Human pericytes (PromoCell, Heidelberg, Germany) and umbilical vein endothelial cells (HuVEC, Lonza, MD, USA) were grown in a cell medium containing growth supplement, in a humidified atmosphere of 95% air and 5% CO_2_ at 37°C. Both cells were used at passages 7–10. For senescent cell experiments, the cells were plated at a density of 3 × 10^5^ cells/well in 6‐well plates and incubated for 3 days. Fresh medium containing 100 nM doxorubicin (DOXO) (Tocris Bioscience, Bristol, UK) was then added to the cells, treated with or without 100 μM PTX, and the cells were cultured for 24 h to induce cellular senescence. The cells were harvested at the end of the treatment for further analysis.

### 2.10 Senescence‐associated β‐galactosidase (SA‐β‐gal) staining

To detect senescent cells, we performed SA‐β‐gal staining using a senescence β‐galactosidase staining kit (Cell Signaling Technology, MA, USA), according to the manufacturer's instructions. Experiments are performed using cells treated with 100 nM DOXO, with or without 100 μM PTX, in a 6‐well plate. Pericytes and HuVECs were rinsed with PBS and fixed in a fixative solution provided with the SA‐β‐gal kit for 15 min at room temperature (25°C). The plates were washed twice with PBS, after which β‐galactosidase staining solution was added, and pericytes and HuVECs were incubated with the staining solution at 37°C without CO_2_ in a dry incubator for 24 h. SA‐β‐gal‐positive cells were detected using light microscopy.

### 2.11 Statistical analysis

Data are expressed as means ± standard error (SE). Differences between the groups were examined for statistical significance using ANOVA with Bonferroni correction (SPSS v. 11.5, IBM, Armonk, NY, USA). A *p* < 0.05 was considered to represent statistical significance.

## 3 RESULTS

### 3.1 Renal function and histological evaluation

Albuminuria, serum creatinine levels, and creatinine clearance were assessed to evaluate the renal function. The aging group showed greater albuminuria than the young group (*p* < 0.05) (Figure 1B). Serum creatinine levels were significantly higher in the aging group than in the young group (*p* < 0.05) (Figure 1C). Although the difference was not statistically significant, creatinine clearance was lower in the aging group than in the young group (Figure 1D). Upon histological examination, the aging group showed a significantly expanded mesangial area and increased tubulointerstitial fibrosis compared to the young group (Figure 1G,H). In the PTX group, albuminuria decreased compared to that in the aging group (*p* < 0.05) (Figure 1B). Serum creatinine decreased and creatinine clearance increased in the PTX group than in the aging group; however, the difference was not statistically significant (Figure 1C). The PTX group showed amelioration of the expanded mesangial area and increased tubulointerstitial fibrosis than in the aging group (*p* < 0.05) (Figure 1G,H).

### 3.2 Immunohistochemistry of collagen I, CD31, and α‐SMA

**FIGURE 2:**
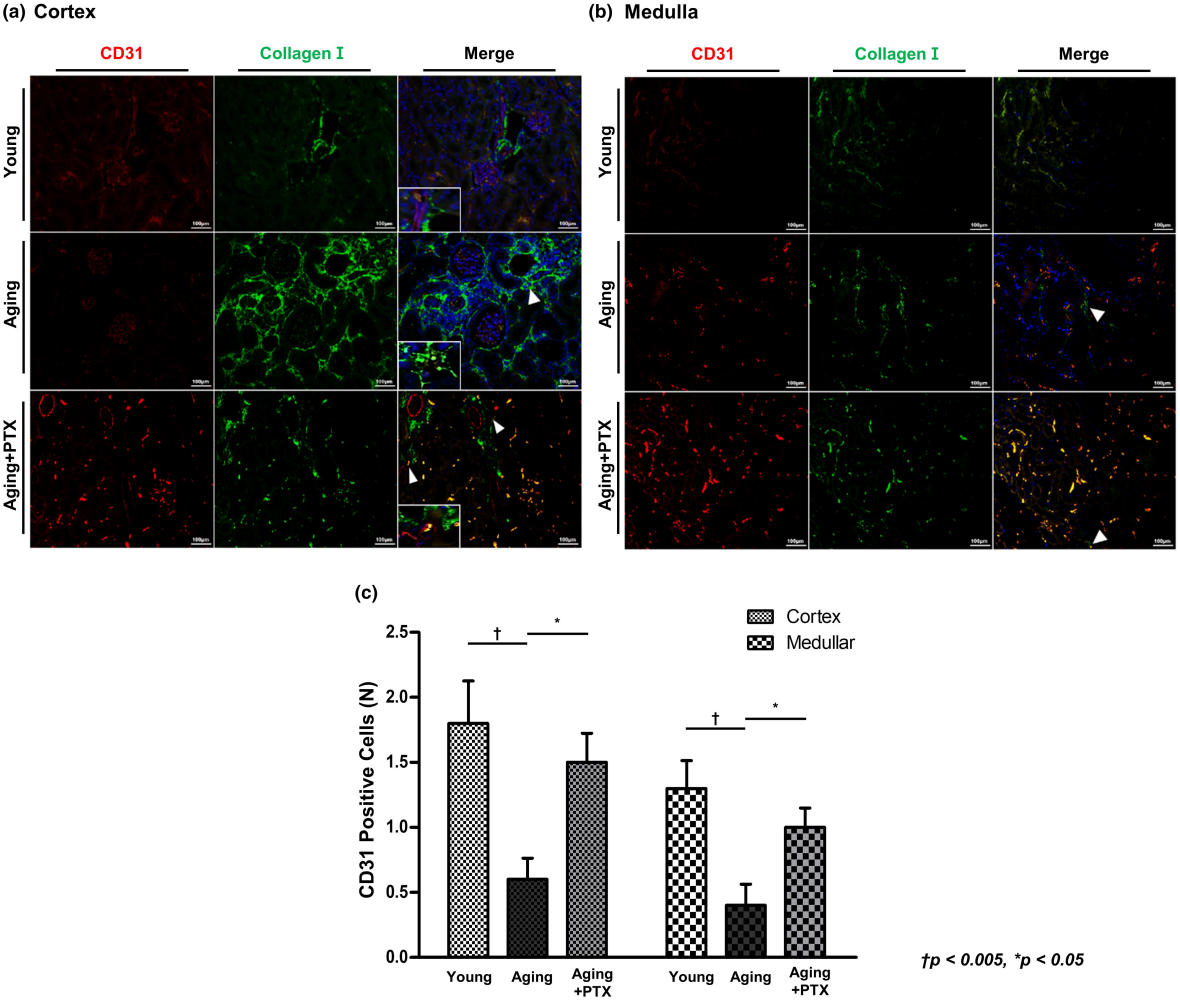
Effects of pentoxifylline (PTX) on interstitial fibrosis and microvascular density. Interstitial fibrosis was assessed by immunofluorescence staining for collagen I (green) and microvascular density was measured by immunofluorescence staining for CD31 (red) in the (a) renal cortex and (b) medulla. (c) Quantitative analyses of CD31‐positive cells. (*N* ≥ 7 for all experiments. ^†^ *p* < 0.005, *p < 0.05).

CD31 staining was used to identify the endothelial cells and the changes in vascular density in the aging kidneys (Figure 2). The number of CD31‐positive cells was compared between the groups in both cortex and medulla. Microvascular density in the aging group was significantly decreased in both cortex and medulla.

**FIGURE 3:**
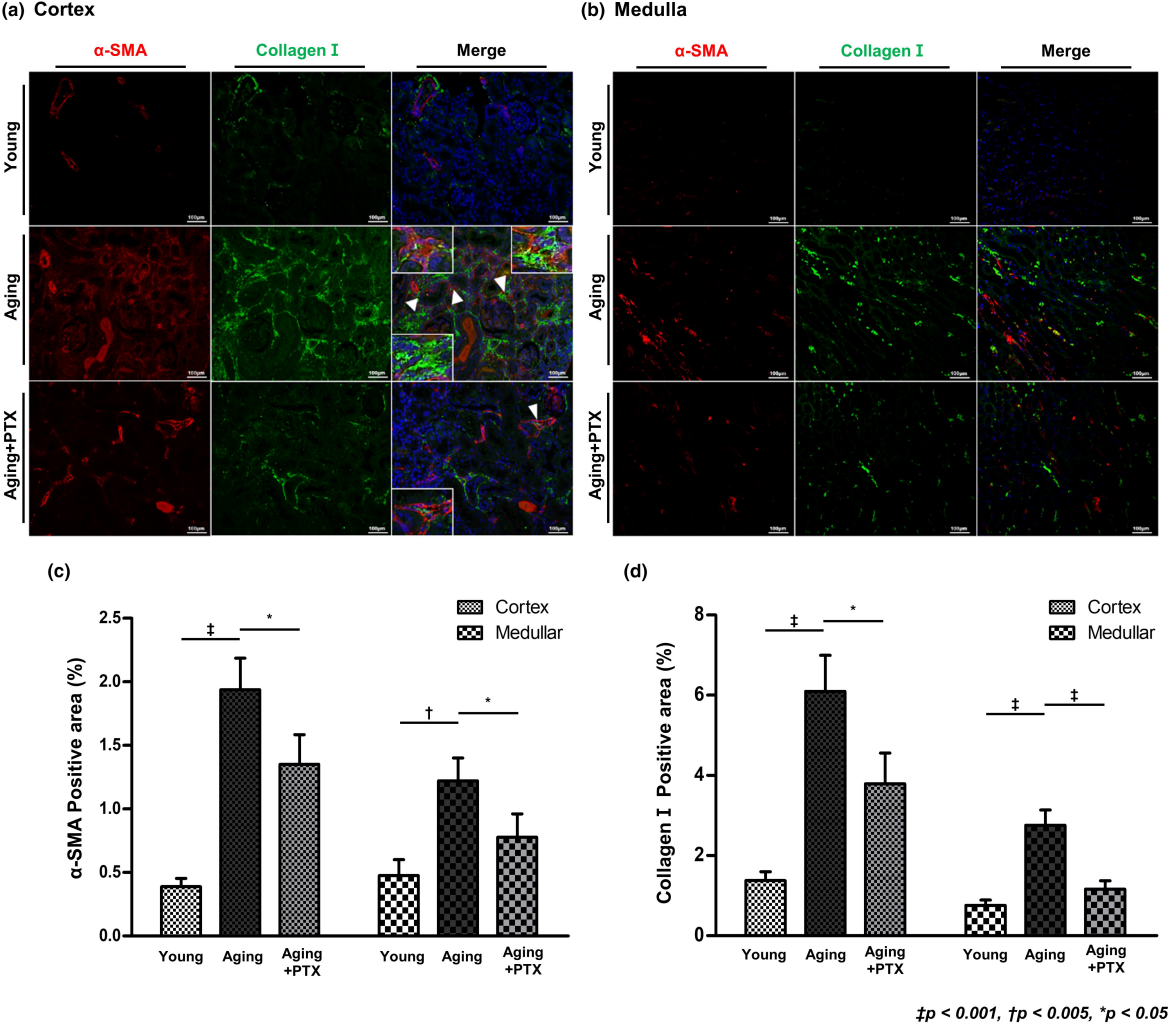
Effects of pentoxifylline (PTX) on interstitial fibrosis and expression of myofibroblasts. Interstitial fibrosis was assessed by immunofluorescence staining for collagen I (green) and myofibroblast was measured by immunofluorescence staining for α‐SMA (red) in (a) renal cortex and (b) medulla. (c, d) Quantitative analyses of the results. (*N* ≥ 7 for all experiments. ^‡^ *p* < 0.001, ^†^ *p* < 0.005, *p < 0.05).

Collagen I expression increased in both cortex and medulla in the aging group compared to that in the young group (Figure 3). The number of cells expressing collagen I was increased in the cortex of aging kidneys. The cells were identified as myofibroblasts by co‐staining with α‐SMA.

### 3.3 Immunohistochemistry for PDGFR‐β and NG2

**FIGURE 4:**
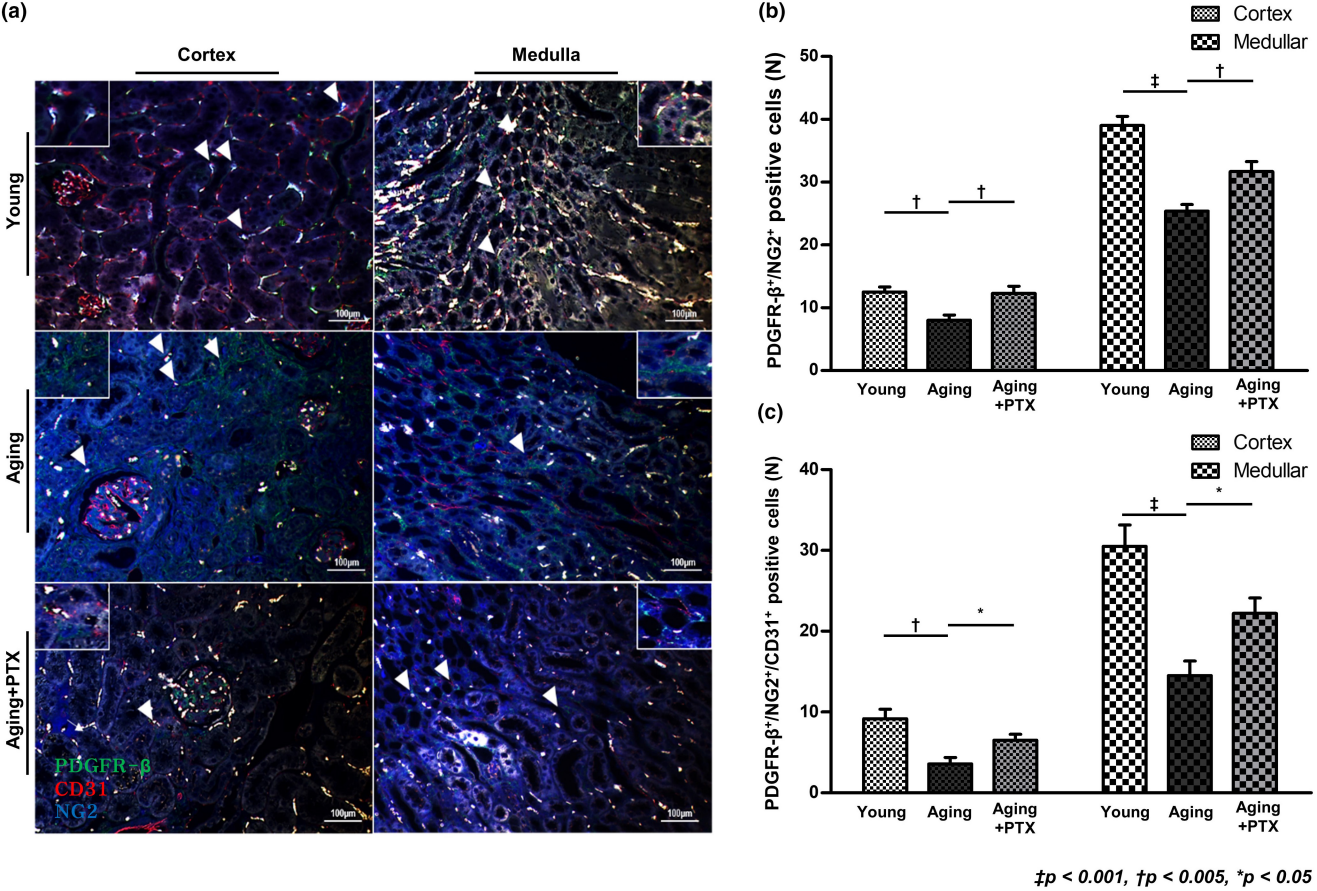
Effects of pentoxifylline (PTX) on interstitial pericytes. (a) Interstitial pericytes were identified by double staining of PDGFR‐β and NG2 (green and blue, respectively) and peritubular capillaries were identified by CD31 staining (red). (b) The number of pericytes was decreased in the aging group compared to that in the young group. Compared to the aging group, PTX group showed increased number of pericytes. (c) The number of pericytes surrounding peritubular capillaries was decreased in the aging group than in the young group. Compared to the aging group, PTX group showed increased number of pericytes surrounding peritubular capillaries. (*N* ≥ 7 for all experiments. ^‡^ *p* < 0.001, ^†^ *p* < 0.005, *p < 0.05).

Pericytes were assessed by co‐staining for PDGFR‐β and NG2. Perivascular location of the pericytes was confirmed by co‐staining with CD31. The number of pericytes in the tubulointerstitium was assessed next (Figure 4). Overall, the number of interstitial pericytes in both the cortex and medulla significantly decreased in the aging group than in young group. Peritubular capillary pericytes also decreased in the aging group. Particularly, the number of medullary peritubular capillary pericytes was significantly reduced in the aging group.

**FIGURE 5:**
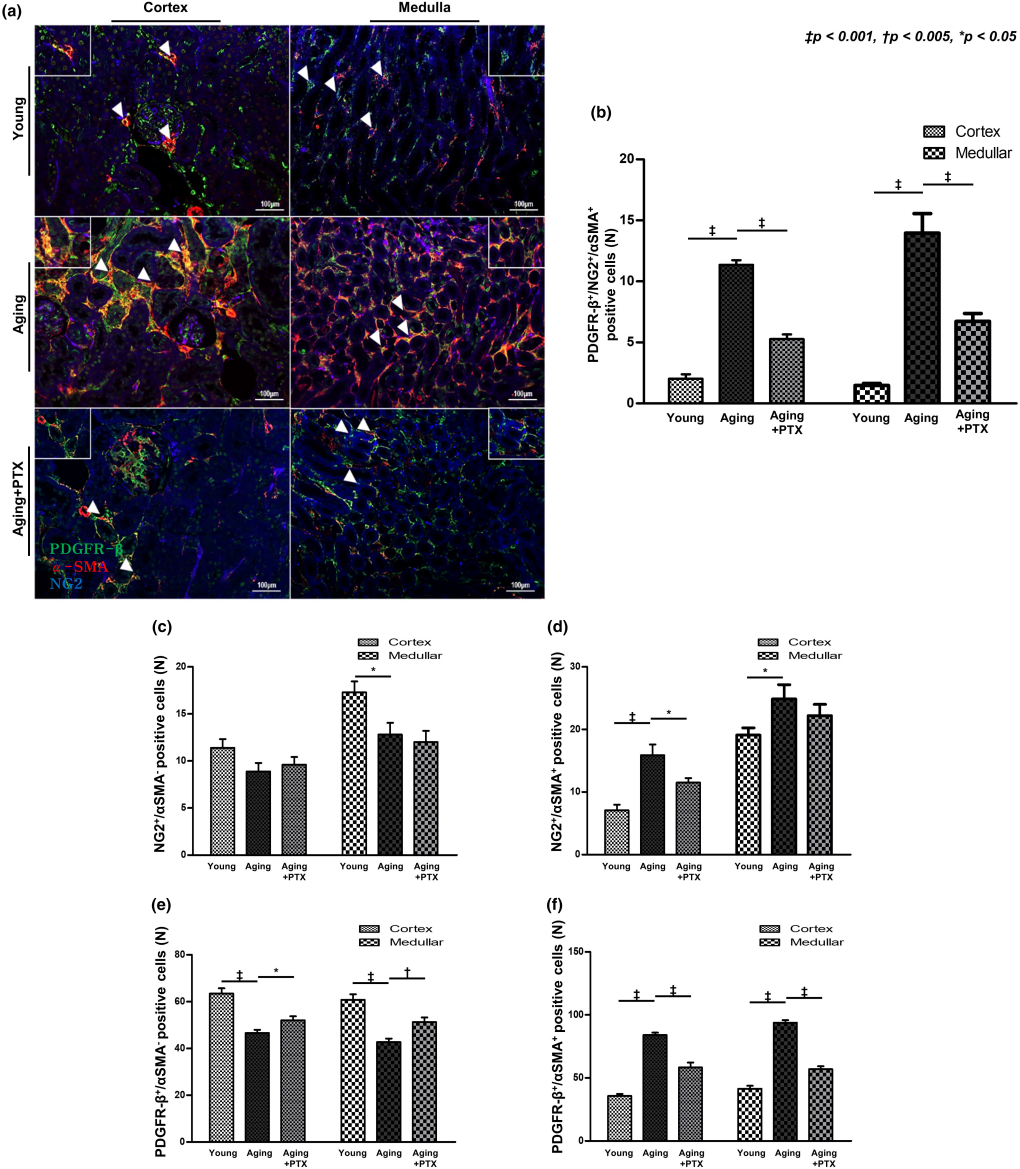
Effects of pentoxifylline (PTX) on the subset of pericytes differentiating into myofibroblasts. (a) Myofibroblasts were identified by α‐SMA staining. Co‐staining of α‐SMA and PDGFR‐β or α‐SMA and NG2 confirmed the presence of myofibroblasts derived from interstitial pericytes. (b–f) Cells with co‐staining of α‐SMA and PDGFR‐β/ α‐SMA and NG2/ α‐SMA, PDGFR‐β, and NG2 were increased in the aging group than in the young group, and decreased in the PTX group than in the aging group. (*N* ≥ 7 for all experiments. ^‡^ *p* < 0.001, ^†^ *p* < 0.005, *p < 0.05).

Renal interstitial pericytes are considered as a source of myofibroblasts (Humphreys et al., 2010). To identify pericyte‐derived myofibroblasts, PDGFR‐β, NG2, and α‐SMA were co‐stained (Figure 5). α ‐SMA‐expressing pericytes were greatly increased in the aging group.

### 3.4 Effect of PTX in aging kidney

The microvascular density was maintained at a level similar to that of the young group, and both collagen I and α‐SMA expression was reduced, indicating that both interstitial fibrosis and myofibroblast numbers were ameliorated with PTX treatment.

The number of overall and peritubular capillary pericytes was higher in the PTX group than in the aging group. The number of α‐SMA‐expressing pericytes was reduced in the PTX group than in the aging group.

**FIGURE 6:**
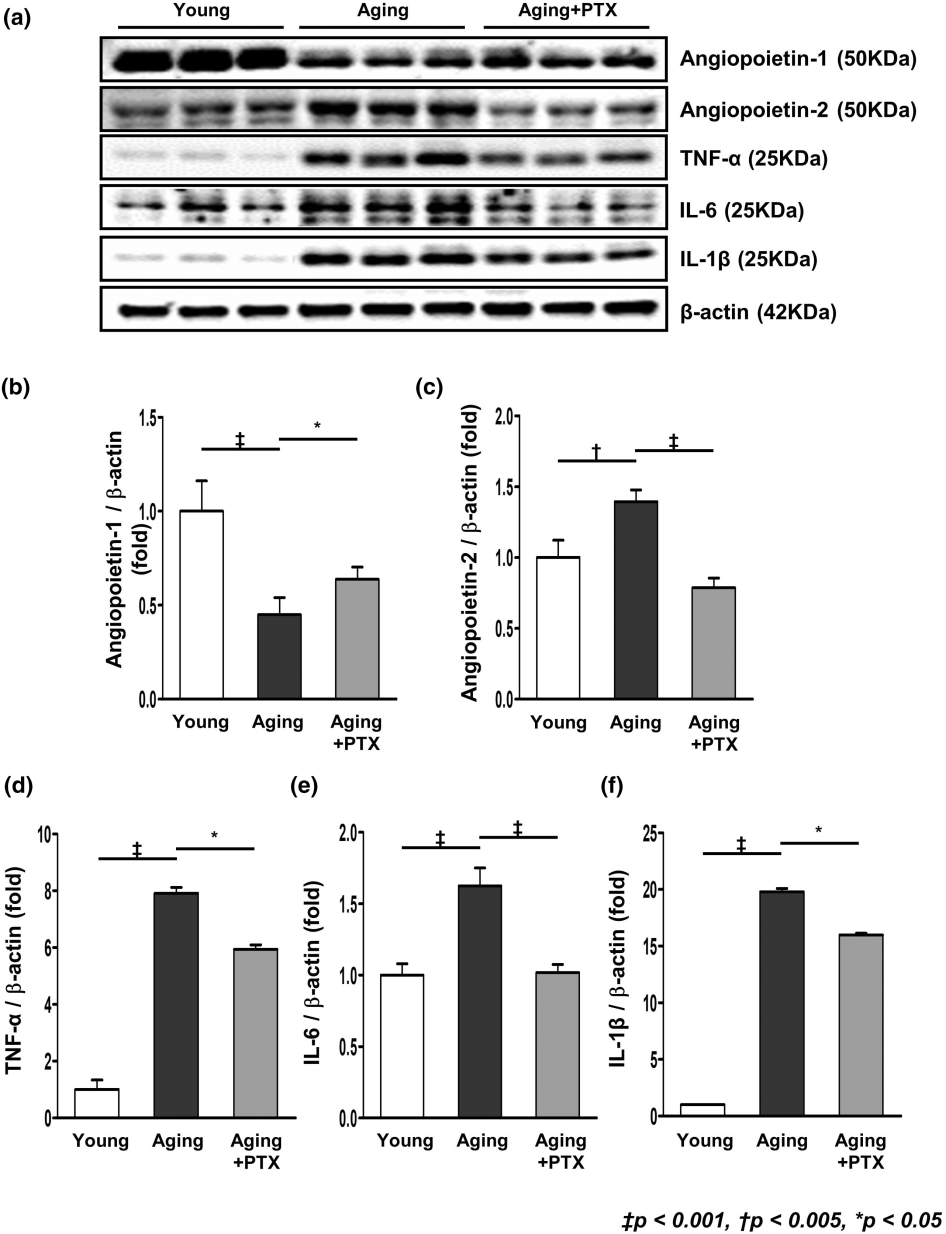
Representative western blots of angiopoietins and inflammatory cytokines. (a) In western blot analysis, PTX group showed increased expression of angiopoietin‐1 and decreased expression of angiopoietin‐2, TNF‐α, IL‐6, and IL‐1β, compared to the aging group. (b–f) Quantitative analyses of the results. (*N* ≥ 7 for all experiments. ^‡^ *p* < 0.001, ^†^ *p* < 0.005, *p < 0.05).

TNF‐α, IL‐6, and IL‐1β were significantly increased in the aging group, and decreased in the PTX group (Figure 6).

### 3.5 Expression of angiopoietin‐1 and angiopoietin‐2

The expression of angiopoietin‐1 decreased while that of angiopoietin‐2 increased in the aging group (Figure 6). On the contrary, expression of angiopoietin‐1 increased and that of angiopoietin‐2 decreased in the PTX group.

### 3.6 Effect of PTX on pericytes and endothelial cells in vitro

**FIGURE 7:**
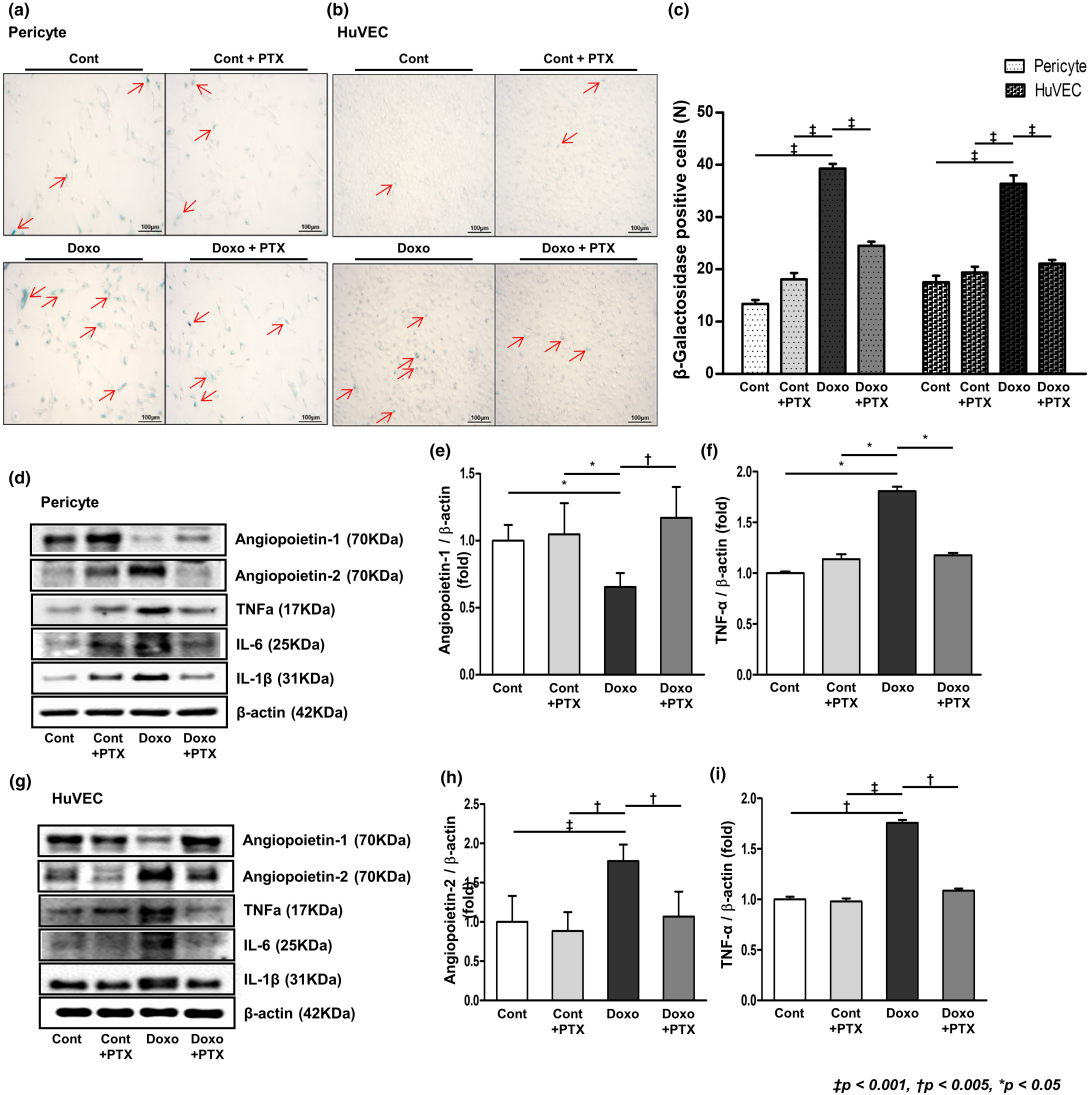
Doxorubicin‐induced cellular senescence and the expression of angiopoietins. Representative images of PTX treatment in (a) senescence‐induced pericytes and (b) HuVECs. (c) Pericytes and HuVECs treated with 200 nM doxorubicin showed increase in the number of β‐galactosidase‐positive cells, which was ameliorated with PTX treatment. (d) Representative western blots demonstrating angiopoietin‐1 and TNF‐α expression in senescence‐induced pericytes. In senescence‐induced pericytes, (e) angiopoietin‐1 expression was increased and (f) TNF‐α expression was decreased with PTX treatment. (g) Representative western blots demonstrating angiopoietin‐2 and TNF‐α expression in senescence‐induced HuVECs. (h, i) In senescence‐induced HuVECs, angiopoietin‐2 and TNF‐α expression was decreased with PTX treatment. (*N* ≥ 7 for all experiments. ^‡^ *p* < 0.001, ^†^ *p* < 0.005, *p < 0.05).

Human pericytes and HuVECs were treated with DOXO to induce in‐vitro senescence. The molecular changes in senile cells were compared to those in the PTX‐co‐administered cells. When assessed by senescence‐associated β‐galactosidase staining, the expression of β‐galactosidase was found increased in the doxorubicin‐treated group, whereas it was ameliorated in the PTX group (Figure 7). In both pericytes and HuVECs, TNF‐α decreased upon PTX treatment. We observed decreased expression of angiopoietin‐1 in senescent pericytes and increased expression of angiopoietin‐2 in senescent HuVECs. Changes in angiopoietin expression were significantly ameliorated in the PTX group.

### 3.7 Changes in renal interstitial pericytes and fibrosis in human aging kidney

Renal interstitial pericytes in kidney tissue, obtained from a kidney transplant donor, were examined to validate the hypothesis that senile changes in pericytes, observed in the experimental model, were consistently observed in humans. The baseline characteristics of donors according to age are presented in Table 1. The mean age of young donors was 24.1 years, whereas that of older donors was 65.2 years. The mean serum creatinine levels were similar between the two groups, at 0.8 mg/dL; however, the mean CKD‐EPI eGFR was 105 mL/min in the young group and 85.6 mL/min in the aging group.

**TABLE 1:** Baseline characteristics.

Variables	Young age (*N* = 12)	Old age (*N* = 12)	*p*‐value
Age (years)	24.1 ± 2.2	65.2 ± 1.7	< 0.0001
Sex (male)	8 (66.7%)	9 (75.0%)	1.000
DM	0 (0%)	0 (0%)	N/A
HTN	0 (0%)	6 (50%)	0.014
BUN (mg/dL)	13.2 ± 1.9	16.3 ± 3.6	0.017
Creatinine (mg/dL)	0.8 ± 0.2	0.8 ± 0.1	0.990
CKD‐EPI eGFR (mL/min/1.73 m^2^)	105.0 ± 19.9	85.6 ± 12.1	0.009
Urine ACR (mg/g Cr)	6.0 ± 5.9	9.8 ± 10.7	0.327

*Note*: Data are expressed as mean ± standard deviation or as number (percentage).

Abbreviations: ACR, albumin‐to‐creatinine ratio; BUN, blood urea nitrogen; DM, diabetes mellitus; eGFR, estimated glomerular filtration rate; HTN, hypertension.

**FIGURE 8:**
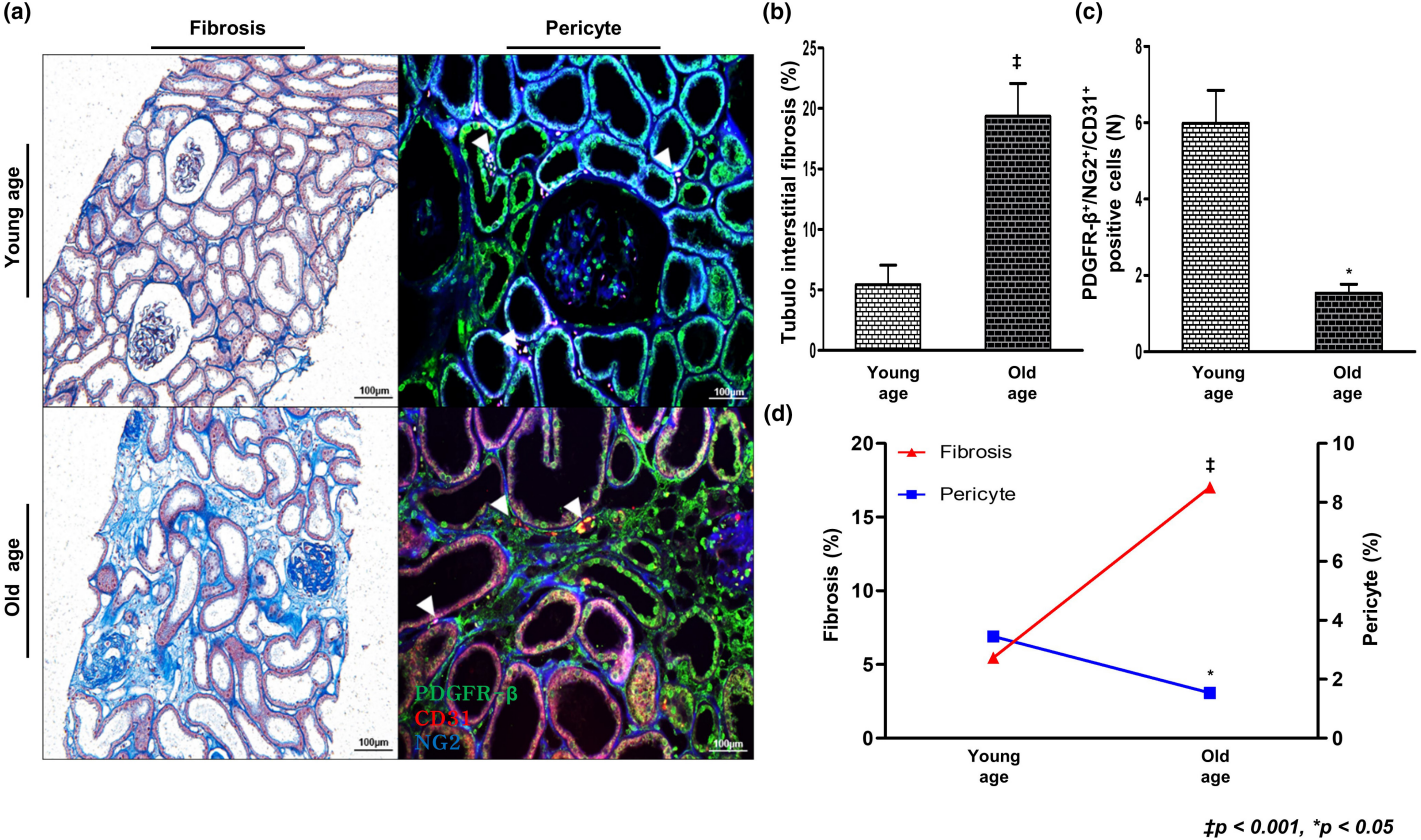
Tubulointerstitial fibrosis and interstitial pericytes in human kidney. (a) Representative sections of the Masson's trichrome‐stained kidney showed significantly less tubulointerstitial fibrosis in the young group than in the aging group (original magnification, 200×). Interstitial pericytes were identified by double staining of PDGFR‐β and NG2 (green and blue, respectively) and peritubular capillaries were identified by CD31 staining (red). (b) Quantitative assessments of the areas of tubulointerstitial fibrosis. (c) The number of pericytes surrounding peritubular capillaries was decreased in the aging group than in the young group. (d) In elderly patients, tubulointerstitial fibrosis was increased and interstitial pericytes were decreased than in the young patients. (*N* = 12 for both groups. ^‡^ *p* < 0.001, *p < 0.05).

Differences in pericytes between young and aging groups were assessed. Significantly more tubulointerstitial fibrotic areas were observed in the aging group than in young group (Figure 8). Peritubular capillary pericytes were identified by the co‐staining of PDGFR‐β, NG2, and CD31. The number of peritubular capillary pericytes was significantly lower in the older age group, and the number of pericytes and the area of tubulointerstitial fibrosis were inversely correlated (Figure S1). Interestingly, there was a difference in the degree of fibrosis even within the young group (Figure S2). In patients with severe fibrosis, the number of pericytes was significantly reduced, even at a young age.

## 4 DISCUSSION

Our previous studies (Jang et al., 2018; Kim et al., 2016, 2018; Lim et al., 2012) had revealed various functional, histological, and molecular changes in aging mouse kidneys. Increased albuminuria, decreased creatinine clearance, increased mesangial volume, and interstitial fibrosis were seen in 24‐month‐old C57/BL6 mice (Kim et al., 2018; Lim et al., 2012). Increased oxidative stress in aging kidney was observed via decreased expression of Sirt1, PGC‐1α, ERR‐1α, PPARα, and Klotho (Lim et al., 2012). In the present study, we observed a decrease in the total number of interstitial pericytes in aging mouse kidneys than in young mouse kidneys. Particularly, a significant reduction in the number of pericytes was observed in the perivascular region. Concurrently, an increase in myofibroblasts, presumed to originate from interstitial pericytes, and subsequent interstitial fibrosis, was observed. The quantitative decline in interstitial pericytes and the loss of perivascular location were significantly attenuated in aging mouse kidneys treated with the PDE inhibitor PTX. Renal interstitial fibrosis and the expression of inflammatory cytokines were also improved following PTX treatment. The characteristic changes in pericytes, observed in aging mouse kidneys, followed a pattern similar to that observed in the human kidney. A significant decrease in angiopoietin‐1 and increase in angiopoietin‐2 were observed in aging mouse kidneys than in young mouse kidneys. However, treatment of aging mouse kidneys with PTX mitigated the changes. Similarly, when human pericytes were subjected to DOXO‐induced senescent injury in vitro, a significant reduction in angiopoietin‐1 expression was observed; when treated with PTX, angiopoietin‐1 expression returned to levels similar to those observed in the control group.

Pericytes are extensively branched mesenchymal cells that surround endothelial cells in the capillary bed and postcapillary venules and are embedded in the capillary basement membrane (Armulik et al., 2011). They form an extensive network around the microvasculature and perform a variety of homeostatic functions related to angiogenesis, vessel maturation, and response to injury (Kramann & Humphreys, 2014). There is no single marker for identifying pericytes; therefore, various markers may be used to identify them, namely PDGFR‐β, NG2, desmin, p75, CD73, and CD248 (Armulik et al., 2011). In our study, we used co‐staining of PDGFR‐β and NG2 to identify pericytes.

Renal interstitial pericytes have recently emerged as key players in the development of renal fibrosis. Detachment of pericytes from perivascular locations and pericyte‐myofibroblast trans‐differentiation result in an increase in renal myofibroblasts, interstitial fibrosis, and capillary rarefaction (Humphreys et al., 2010; Kramann et al., 2013). Stefanska et al (Stefanska et al., 2015) reported the decline in renal interstitial pericytes as a critical step in the development of changes in peritubular vasculature with aging, and accompanying fibrosis in an aging mouse model. There have also been several studies on the association between pericytes and age‐related senile changes in organs other than the kidneys. Berthiaume et al. (2022) had reported that pericyte remodeling is slower in aged brain. Defective pericyte remodeling results in persistent capillary dilatation and disruption of capillary flow and structure. Nag et al. (2021) had reported that pericyte damage leads to endothelial changes and loss of choriocapillaris in aged humans. A study conducted through the isolation of human renal pericytes reported an association between pericytes and the expression and secretion of renin (Stefanska et al., 2016). However, research regarding the role or alterations of pericytes in human aging kidneys or renal diseases has not been reported till date. Our study showed that characteristic findings, including a quantitative decline in interstitial pericytes, in aging kidneys of animal models, are evident in the kidneys of elderly patients.

Pentoxifylline is a very safe nonselective PDE inhibitor and has various effects against cell proliferation, inflammation, and extracellular matrix accumulation (Lin et al., 2004). PTX inhibits PDE 1–5, and has long been used to treat peripheral vascular diseases. After the 1990s, PTX has emerged as an inflammatory modulator. A study by Vadiei et al. (1996) suggested that PTX can prevent acute kidney injury by reducing vascular congestion and inhibiting nitric oxide release from activated macrophages. Since the 2000s, various beneficial effects of PTX, including its anti‐inflammatory actions, have gained attention. Numerous clinical studies have primarily focused on diabetic kidney disease (DKD); (Donate‐Correa et al., 2022) the notable PTX for Renoprotection in Diabetic Nephropathy (PREDIAN) study (Navarro‐González et al., 2015) investigated 169 registered patients with DKD. It revealed that patients receiving PTX treatment exhibited a lower eGFR decline and reduced albuminuria than those in the control group. However, studies on the effects of PTX on aging are still limited. Wang et al.'s (2021) study reported that PTX improved cognitive deficits in aging mice through Nrf2/PGC‐1a activation; in our study, PTX improved proteinuria, tubulointerstitial fibrosis, and microvascular rarefaction in aging mouse kidney.

Angiopoietin is a member of vascular growth factor family. There are several types of angiopoietins, including angiopoietin‐1 and angiopoietin‐2. Angiopoietin‐1 is known to improve endothelial survival and contribute to pericyte stabilization through the upregulation of Tie‐2 signaling, whereas angiopoietin‐2 is known to antagonize this action (Brindle et al., 2006). Recent studies have indicated the association of the angiopoietin/Tie‐2 signaling pathway with the development of atherosclerosis (Hayashi et al., 2020). Pöss et al. (2015) reported the plasma levels of angiopoietin‐2 as an independent predictor of mortality in patients with acute myocardial infarction. Chang et al. (2014) revealed, in a cross‐sectional cohort study, that high plasma levels of angiopoietin‐2 are associated with worsened arterial stiffness and increased cardiovascular risk in patients with chronic kidney disease. Additionally, in an in‐vitro study, Cai et al. (2008) had reported that angiopoietin‐1 improved the survival and activation of retinal pericytes, whereas angiopoietin‐2 increased pericyte apoptosis. In this study, we observed decreased angiopoietin‐1 expression and increased angiopoietin‐2 expression in the kidneys of aging mice. Based on previous findings, the increased expression of angiopoietin‐2 in aging mouse kidneys may contribute to the upregulation of profibrotic and proinflammatory cytokines in endothelial cells and the decrease in interstitial pericytes.

Venneri et al. (2019) revealed that angiopoietin‐1 is defective in mouse and human models of diabetes and is normalized by PDE 5 inhibition. They claimed that PDE 5 inhibition results in modulation of the angiopoietin/Tie2 signaling axis and vessel stabilization. In the present study, PTX treatment was found to be associated with increased angiopoietin‐1 expression in the kidneys of aging mice. As a non‐selective PDE inhibitor, PTX could modulate angiopoietin/Tie2 signaling, leading to an increase in angiopoietin‐1 expression and a decrease in angiopoietin‐2. Inflammatory mediators may also be involved in angiopoietin signaling and expression. Research by Kim et al. (2000) indicated that TNF‐ α increases angiopoietin‐2 expression in human endothelial cells, and another study (Fan et al., 2004) reported that both TNF‐ α and IL‐1β decrease angiopoietin‐1 expression in human endothelial cells. Furthermore, other studies suggested that IL‐6 promotes VEGF expression and leads to the downregulation of angiopoietin‐1 expression while upregulating angiopoietin‐2 expression in endothelial cells (Kayakabe et al., 2012). In our study, direct inhibition of PDE by PTX showed anti‐inflammatory effects and alleviated age‐related changes in the PTX‐treated aging kidney. This renoprotective effect of PTX may be contribute to the preservation of perivascular pericytes.

In conclusion, our study reported that the PDE inhibitor PTX improved renal interstitial fibrosis and inflammation in aging mouse kidneys. The protective effects were suggested to be associated with the amelioration of pericytes reduction and the transition to myofibroblasts following phosphodiesterase inhibition. Additionally, the upregulation of angiopoietin‐1 expression may exert direct or indirect potential impacts on these processes. To the best of our knowledge, this is the first report on changes in renal interstitial pericytes in aging human kidneys.

## AUTHOR CONTRIBUTIONS

Hyung Duk Kim, Eun Nim Kim, Ji Hee Lim, Yaeni Kim, Tae Hyun Ban, Hajeong Lee, Yon Su Kim, Cheol Whee Park, and Bum Soon Choi designed and conceptualized the study. Hyung Duk Kim, Eun Nim Kim, and Ji Hee Lim conducted experiments. Hyung Duk Kim, Eun Nim Kim, Cheol Whee Park, and Bum Soon Choi analyzed the data. Eun Nim Kim made the figures. Hyung Duk Kim, Eun Nim Kim, and Bum Soon Choi wrote the manuscript. All of the authors critically analyzed the manuscript and agreed to its final version for publication.

## FUNDING INFORMATION

This study was supported by the Basic Science Research Program through the National Research Foundation of Korea (NRF), funded by the Ministry of Education (NRF‐2019R1I1A1A01063370), and the Alumni of The Catholic University of Korea Division of Nephrology grant.

## CONFLICT OF INTEREST STATEMENT

The authors declare that the research was conducted in the absence of any commercial or financial relationships that could be construed as a potential conflict of interest.

## Supporting information


Figure S1.

## Data Availability

The data presented in this study are available in the article. Further inquiries can be directed to the corresponding author.
