# Supplementary material for: Phosphodiesterase inhibitor ameliorates senescent changes of renal interstitial pericytes in aging kidney
Source: Aging Cell. 2023 Dec 28;23(3):e14075. doi: 10.1111/acel.14075 (PMC10928568; doi:10.1111/acel.14075)
Supplement: Supplementary file 1 — Figure S1. [file ACEL-23-e14075-s001.pdf]

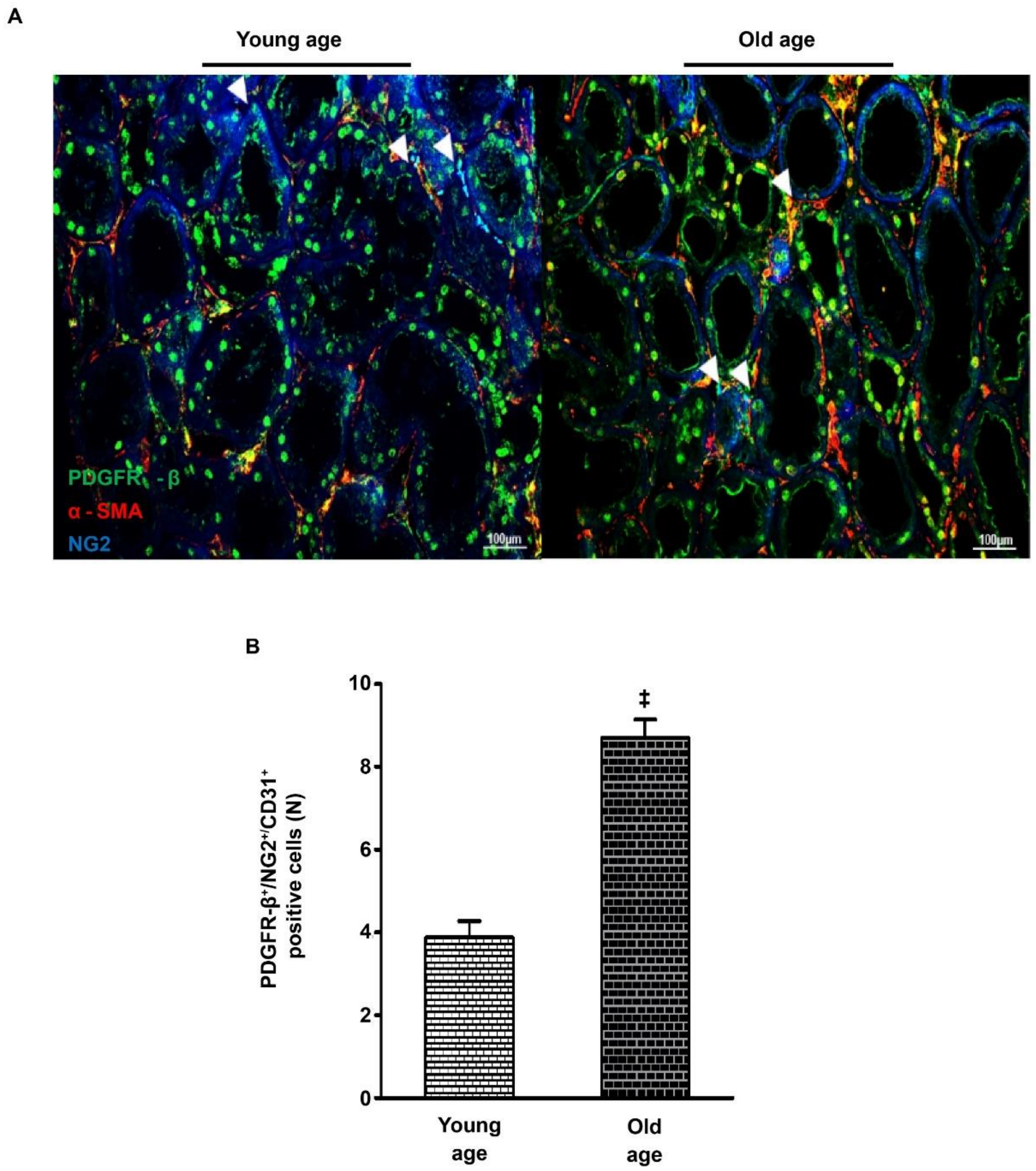

Supplemental Figure 1. Tubulointerstitial fibrosis and the subset of pericytes differentiating into myofibroblasts in human kidney. (A) Myofibroblasts were identified by α-SMA staining. Co-staining of α-SMA, PDGFR-β, and NG2 confirmed the presence of myofibroblasts derived from interstitial pericytes. (B) Cells with co-staining of α-SMA, PDGFR-β, and NG2 were increased in the aging group than in the young group. ($N = 12$ for both groups. ‡$p < 0.001$)

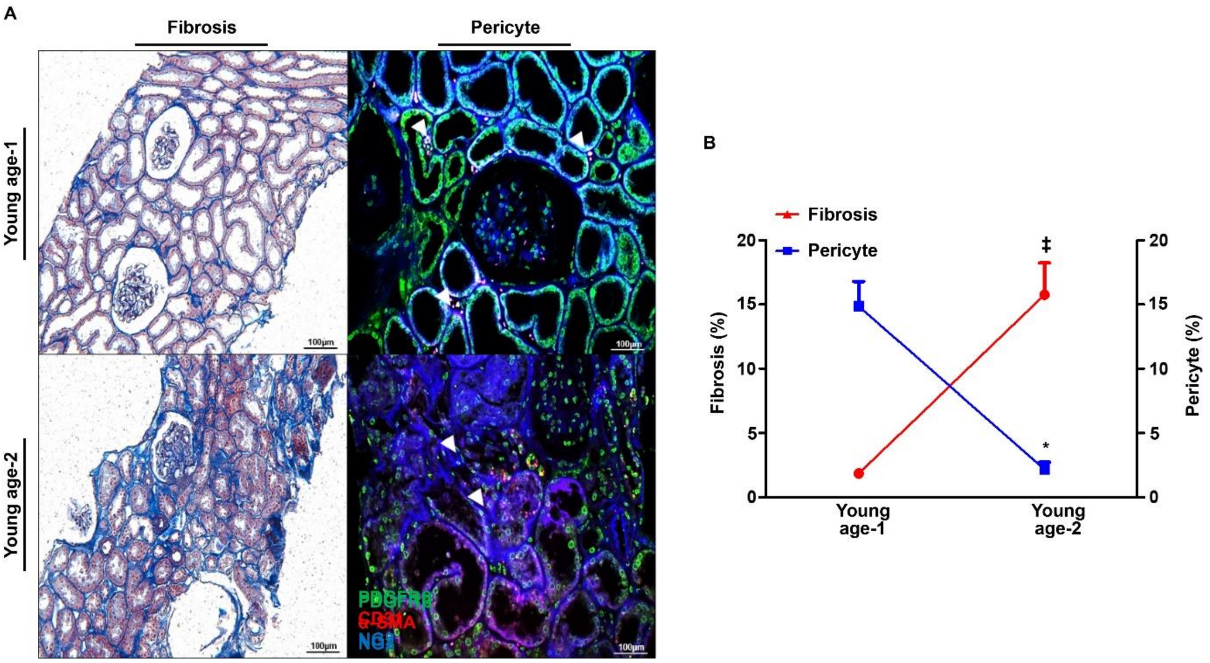

Supplemental Figure 2. Individual differences in terms of tubulointerstitial fibrosis and interstitial pericytes in young human kidneys. (A) Representative sections of the Masson's trichrome-stained kidney showing tubulointerstitial fibrosis (original magnification, 200×). Interstitial pericytes were identified by double staining of PDGFR-β and NG2 (green and blue, respectively), and peritubular capillaries were identified by CD31 staining (red). (B) Among the young patients, those with more severe tubulointerstitial fibrosis exhibited a decreased number of interstitial pericytes. (‡$p < 0.001$)
